# Construction and validation of a 28-day mortality prediction model for refeeding syndrome in critically ill patients

**DOI:** 10.3389/fnut.2026.1888609

**Published:** 2026-09-25

**Authors:** Jiajia Pan, Xuanle Wu, Nina Wang, Guangfa Wei, Yuyan Zhang, Qining Zheng, Min Zhang, Weili Liu

**Affiliations:** Department of Intensive Care Unit, Affiliated Hospital of Yangzhou University, Yangzhou, Jiangsu, China

**Keywords:** 28-day mortality, critical illness, machine learning, prediction model, refeeding syndrome

## Abstract

**Background and aims:**

Refeeding syndrome (RFS) is a common and serious metabolic reaction during nutritional support in critically ill patients, associated with substantially increased mortality risk and poor prognosis. Existing studies mainly focus on predicting its occurrence, while predictive models for 28-day mortality risk remain lacking. This study aimed to develop and validate an interpretable machine learning model to support short-term prognostic assessment in clinical practice.

**Methods:**

Data for critically ill patients with RFS were extracted from the Medical Information Mart for Intensive Care IV (MIMIC-IV, 2008–2019) as the internal cohort. A total of 8,705 patients were randomly assigned to training and test sets at a 7:3 ratio. Thirty-eight candidate variables were initially included, and feature selection was performed using least absolute shrinkage and selection operator (LASSO) logistic regression. Three machine learning models were subsequently constructed, and the optimal model was validated using two independent datasets. Model interpretability was enhanced using Shapley additive explanations (SHAP). Kaplan–Meier curves were used to compare survival between the high- and low-risk, and nomograms were constructed to facilitate clinical application.

**Results:**

Nine variables were ultimately selected to construct the logistic regression model. The model demonstrated excellent discriminative performance and generalizability, with area under the receiver operating characteristic curve (AUC) values of 0.821, 0.809, and 0.731 in the internal test set, external validation set I (MIMIC-III), and external validation set II (the Affiliated Hospital of Yangzhou University), respectively. SHAP analysis identified partial pressure of oxygen, mean blood glucose level, and age as the key prognostic factors and further revealed nonlinear effects among the variables. Kaplan–Meier survival curves confirmed that the model significantly distinguished between the high- and low-risk groups across all cohorts (all *p* < 0.05). Decision curve analysis showed that the model provided favorable clinical net benefit in both external validation cohorts.

**Conclusion:**

The interpretable machine learning model developed in this study effectively predicts the 28-day mortality risk of RFS in critically ill patients, provides an objective reference for individualized clinical decision-making, and may help improve patient prognosis.

## 1 Introduction

The concept of refeeding syndrome (RFS) dates back to the 1940s, when chronically starved prisoners of war died prematurely after ingesting large amounts of calories; these reports are often cited as basis for the modern recognition of RFS (1).

RFS is characterized by metabolic and electrolyte disturbances that occurs following the reintroduction of nutrition after a period of deprivation and may result in respiratory failure, cardiac dysfunction, neurological disorders, and death (2).

In 2020 the American Society for Parenteral and Enteral Nutrition (ASPEN) defined RFS as a condition occurring within 5 days of initiating or substantially increasing energy intake, in which serum phosphate, potassium or magnesium (either individually or in combination) decrease by at least 10% from baseline and/or are accompanied by organ dysfunction related to thiamine deficiency (3).

However, challenges in early recognition and the absence of universally accepted diagnostic criteria (4) have contributed to a high reported prevalence of RFS (up to 52.5%), with associated mortality rates ranging from 15.6 and 83.3% (5). A meta-analysis further demonstrated that the risk of death within 1 month is 27% higher in patients with RFS than in those without RFS (6).

In recent years, the population at risk for refeeding syndrome has expanded beyond wartime starvation groups to encompass modern clinical settings, where it is now recognized as a common and easily overlooked complication across multiple departments (4). Compared with general hospitalized patients, those admitted to the intensive care unit (ICU) represent a core population at high risk for RFS. Prolonged physiological stress, severe malnutrition, impaired metabolic regulation, and extended fasting are common in ICU patients, and manifestations in RFS may be masked by underlying critical illnesses further complicating early recognition (7).

However, in current clinical practice, existing assessment tools tend to focus on the diagnosis of RFS itself, and criteria for high-risk stratification in critically ill patients have not yet been harmonized across guidelines, including those from ASPEN (3), the National Institute for Health and Clinical Excellence (NICE) (8), modified NICE criteria (9), and standards developed by the Rio group (10). This lack of consensus makes it difficult to identify patients at high risk of death in advance and to implement targeted interventions. Accordingly, this study aimed to develop a predictive model for 28-day mortality among critically ill patients with refeeding syndrome (RFS) using the Medical Information Mart for Intensive Care IV (MIMIC-IV) database. The model was subsequently subjected to dual external validation using the MIMIC-III database (2001–2007) and an ICU clinical dataset from the Affiliated Hospital of Yangzhou University. Through diversified validation across databases and clinical centers, this study sought to further enhance the model’s generalizability and clinical applicability, thereby providing a scientifically robust and reliable decision-support tool for the early identification of patients at high risk of RFS-related mortality and for the implementation of individualized nursing interventions.

## 2 Materials and methods

### 2.1 Data sources

This retrospective cohort study used data from two large open-access databases and one local clinical validation cohort. First, patient data admitted between 2008 and 2019 were extracted from MIMIC-IV (v3.1) for model development and internal validation, and data admitted between 2001 and 2007 were extracted from MIMIC-III (v1.4) as the first external validation set to ensure the preliminary generalizability of the model. The MIMIC database was developed by the Massachusetts Institute of Technology (MIT) and Beth Israel Deaconess Medical Center (BIDMC) (11). Second, real-world clinical data were collected from the Department of Critical Care Medicine of the Affiliated Hospital of Yangzhou University as the second external validation set to evaluate the practical performance of the model in a local healthcare setting. Regarding privacy protection and ethical compliance, the MIMIC database was approved by the Institutional Review Board of the Massachusetts Institute of Technology (MIT), and all data were rigorously de-identified in accordance with the Medical Data Privacy Protection Specification (12). The researcher (Xuanle Wu) completed the required training and received authorization to access the database (Record ID: 13748561).

### 2.2 Study population and definitions

Baseline clinical data for critically ill patients were extracted from the MIMIC database using Navicat Premium (version 17.0) with Structured Query Language (SQL), indexed by each patient’s unique identifier (subject_id). For patients with multiple ICU admissions, only the first ICU admission during the first hospitalization was included in the analysis (13). For the modeling cohort, patient information from MIMIC-IV between 2008 and 2019 was extracted. For the external validation cohorts, patient information from MIMIC-III between 2001 and 2007 and ICU patient data from the Affiliated Hospital of Yangzhou University between April 5, 2025, and April 6, 2026, were extracted. All patients in the hospital-based validation cohort had complete 28-day outcome data, which were ascertained through the hospital electronic medical record system and telephone follow-up.

The diagnosis of RFS strictly followed the 2020 ASPEN criteria. Patients were considered to have RFS if, within 5 days after initiating or increasing energy intake, serum phosphate, potassium, or magnesium levels decreased by ≥10% compared with pre-refeeding values (3).

The inclusion criteria were: (1) age ≥18 years; (2) first ICU admission; and (3) receipt of enteral nutrition support for ≥3 days during the ICU stay. The exclusion criteria were: (1) pregnancy or lactation; (2) incomplete clinical data; and (3) conditions associated with hypophosphatemia, including (a) continuous renal replacement therapy, (b) hemodialysis, (c) parathyroidectomy within the previous 3 months, (d) advanced malignant tumors, (e) hepatic insufficiency, and (f) treatment for hyperphosphatemia. The patient screening process is shown in Figure 1.

**Figure 1:**
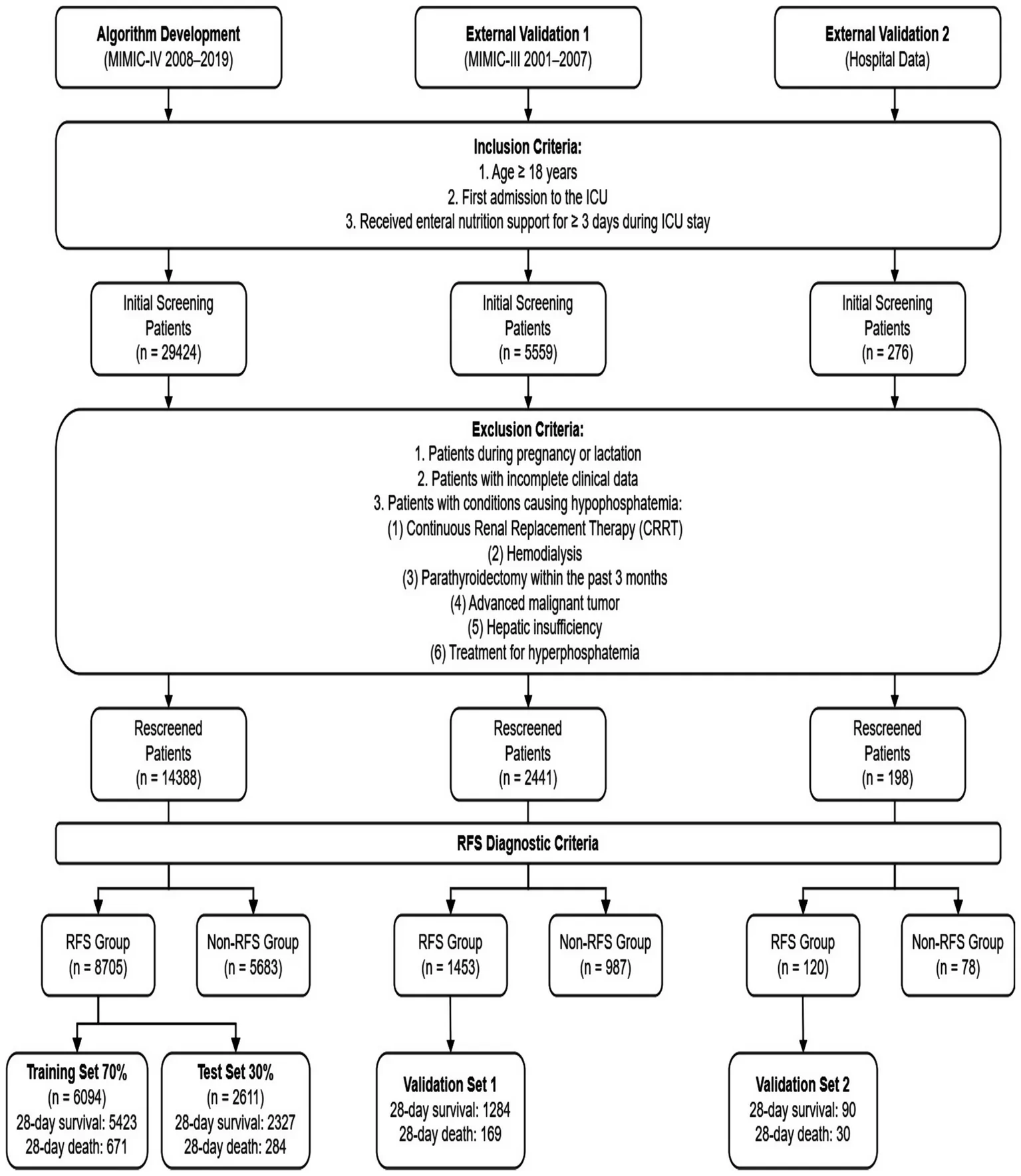
Patient screening flow chart.

### 2.3 Data collection

The primary outcome of this study was 28-day all-cause mortality in critically ill patients who developed refeeding syndrome. Based on a preliminary literature review and expert consultation, 41 clinical variables were initially included and categorized as follows: Demographic characteristics, clinical scores, comorbidities, and laboratory indicators intended to reflect the patients’ pre-feeding status were collected at ICU admission or, when repeated measurements were available, using the most recent value obtained before the initiation of enteral nutrition. Treatment- and clinical course-related variables were extracted from the corresponding ICU records.

(1) Demographics: age, gender, body mass index (BMI), and ethnicity;

(2) Clinical scores: Sequential Organ Failure Assessment (SOFA), Glasgow Coma Scale (GCS), Acute Physiology and Chronic Health Evaluation II (APACHE II), and modified Nutritional Risk in the Critically Ill (mNUTRIC) score. Because interleukin-6 was unavailable in the MIMIC databases, the mNUTRIC score rather than the original NUTRIC score was used.

(3) Laboratory indicators: serum phosphorus, magnesium, potassium, calcium, sodium, hemoglobin, lactate, creatinine, urea nitrogen, partial pressure of carbon dioxide, partial pressure of oxygen, acid–base status, activated partial thromboplastin time, international normalized ratio, glycated hemoglobin, B-type natriuretic peptide, albumin, globulin, total protein, total bilirubin, C-reactive protein, D-dimer, mean glucose value in the ICU prior to feeding, and the coefficient of variation (CV) of blood glucose. The CV for blood glucose was calculated as: CV = (standard deviation/mean blood glucose) × 100%. For all laboratory variables except glucose-related indices, the most recent test results obtained before initiation of nutritional support were used;

(4) Comorbidities and nutritional status: hypertension, diabetes mellitus, heart failure, myocardial infarction, and malnutrition; and

(5) Clinical course and treatment-related variables: feeding within 48 h of ICU admission, hospital stay before ICU admission, length of ICU stay, and duration of mechanical ventilation.

The initiation time of enteral nutrition was available and was used to identify the pre-feeding laboratory measurements. Because this was a retrospective observational study, enteral nutrition and electrolyte management followed routine individualized clinical practice rather than a standardized study protocol. In the hospital cohort, serum phosphate, potassium, and magnesium were generally reassessed every 4–12 h, whereas monitoring intervals varied in the database-derived cohorts; detailed information on the initial caloric dose, feeding advancement or interruption, and electrolyte replacement agents and doses was not consistently available.

### 2.4 Data extraction and processing

Missing data were present in the database (14). To minimize potential model bias, variables with more than 30% missing values (15) were excluded (see Supplementary Figure S1), and the remaining 32 variables were included for subsequent imputation. The dataset was randomly divided into a training set and an internal test set at a 7:3 ratio using stratification based on the 28-day mortality outcome.

Multiple imputation was performed on the training set using the *mice* package (16) to ensure the plausibility and robustness of imputed values. Predictive mean matching (PMM) was used as the core imputation method, with five imputations generated over a maximum of 10 iterations. A fixed random seed (123) was applied to ensure reproducibility. Missing values in the test set were imputed based on the statistical characteristics of the training set to maintain consistency in the imputation strategy.

Univariate analyses were conducted in the training set with 28-day mortality as the outcome, and 24 variables with *p* < 0.1 were retained for further analysis (Supplementary Table S1). These variables were subsequently entered into least absolute shrinkage and selection operator (LASSO) logistic regression, which reduces model complexity and overfitting by shrinking less informative coefficients toward zero (17). Ultimately, nine core variables with non-zero coefficients were identified.

To confirm the independent associations between these nine core variables and 28-day mortality among critically ill patients with RFS, they were further included in a multivariable logistic regression analysis. If the regression coefficients of all nine variables achieved *p* < 0.05, these variables were considered independent predictors of 28-day mortality among critically ill patients with RFS and were retained as candidate features for subsequent prediction model construction.

### 2.5 Model development and performance evaluation

Three machine learning models [logistic regression (LR), random forest (RF), and extreme gradient boosting (XGBoost)] were developed to predict 28-day mortality in critically ill patients. Models were trained using the training set and evaluated in the internal test set and external validation set. Model performance was assessed using the area under the receiver operating characteristic curve (AUC), sensitivity, specificity, Youden index, accuracy, positive predictive value (PPV), negative predictive value (NPV), and F1 score. Calibration curves were used to assess agreement between predicted and observed outcomes (18), and clinical utility was evaluated using decision curve analysis (DCA) (19).

Model performance was also compared with commonly used clinical scoring systems, including the combined SOFA+APACHE II score, APACHE II score alone, and SOFA score alone. The model with the best overall predictive performance was selected based on a comprehensive evaluation of these metrics and was further validated using the MIMIC-III database and the hospital dataset to assess its generalizability.

To interpret the contribution of the 9 core features to model predictions, Shapley Additive Explanations (SHAP) were applied, allowing quantitative assessment of each feature’s impact on the predicted outcome (20). Kaplan–Meier survival curves combined with the log-rank test (21) were used to stratify patients into high- and low-risk groups based on the optimal threshold of the LR model and to evaluate differences in 28-day survival between the two groups.

### 2.6 Statistical analysis

All continuous variables exhibited skewed distributions and are presented as medians with interquartile ranges [Md(P25, P75)]. Group comparisons were performed using the Mann–Whitney *U* test. Categorical variables were presented as counts and proportions (*n*, %) and were compared using the chi-square test or Fisher’s exact test, as appropriate. All statistical tests were two-tailed, and *p* < 0.05 was considered statistically significant. Multicollinearity among predictors was assessed using variance inflation factors (VIFs) and tolerance values. A VIF > 5 was considered indicative of multicollinearity (22). All statistical analyses, model development, and performance evaluation were conducted using R software (version 4.5.2) with the following packages: *tableone*, *caret*, *mice*, *glmnet*, *pROC*, *rms*, *rmda*, *survival*, *shapviz* and *shiny*.

## 3 Results

### 3.1 Comparison of baseline characteristics

A total of 8,705 critically ill patients who developed refeeding syndrome were screened based on the inclusion and exclusion criteria, and were divided into a training set (*n* = 6,094) and a test set (*n* = 2,611). Stratified random sampling based on 28-day ICU mortality was used to ensure a consistent outcome distribution for model development and internal validation. The comparison of baseline characteristics between the two groups is shown in Table 1.

**Table 1:** Comparison of baseline characteristics of ICU patients between training and test sets.

Variables	Training set (*n* = 6,094)	Test set (*n* = 2,611)	*p*-value
Age (years)	66(55, 76)	67(56, 76)	0.403
Gender, *n* (%)			0.127
Male	3,828(62.8)	1,685(64.5)	
Female	2,266(37.2)	926(35.5)	
BMI (kg/m^2^)	27.89(24.22, 32.36)	27.75(24.23, 32.28)	0.589
Ethnicity, *n* (%)			0.911
Asian	172(2.8)	64(2.5)	
Black	427(7.0)	184(7.0)	
Latinos	194(3.2)	82(3.1)	
Caucasian	3,727(61.2)	1,607(61.5)	
Other	1,574(25.8)	674(25.8)	
SOFA score	5(3, 7)	5(3, 7)	0.094
GCS score	15(14, 15)	15(14, 15)	0.465
APACHE II score	17(13, 22)	17(13, 22)	0.118
mNUTRIC score	4(3, 6)	5(3, 6)	0.102
Magnesium (mmol/L)	2.1(1.9, 2.4)	2.1(1.9, 2.4)	0.312
Phosphorus (mmol/L)	3.50(3.0, 4.2)	3.50(3.05, 4.10)	0.928
Potassium (mmol/L)	4.25(3.93, 4.57)	4.25(3.95, 4.6)	0.251
Hemoglobin (g/dL)	10.5(9.0, 12.2)	10.5(9.0, 12.2)	0.962
Total calcium (mmol/L)	8.2(7.8, 8.7)	8.2(7.8, 8.6)	0.997
Lactate (mmol/L)	1.9(1.3, 2.7)	1.9(1.3, 2.7)	0.848
Creatinine (mg/dL)	0.9(0.7, 1.2)	0.9(0.7, 1.2)	0.668
Urea nitrogen (mg/dL)	17.0(12.0, 24.0)	17.0(13.0, 24.0)	0.244
Partial pressure of carbon dioxide (mmHg)	42.0(37.0, 47.0)	42.0(37.0, 47.0)	0.794
pH	7.37(7.32, 7.42)	7.37(7.32, 7.42)	0.901
Partial pressure of oxygen (mmHg)	177.0(84.0, 312.8)	174.0(86.5, 312.0)	0.649
Activated partial thromboplastin time (s)	29.6(26.6, 34.6)	29.8(26.5, 34.4)	0.806
International normalized ratio	1.3(1.2, 1.5)	1.3(1.1, 1.5)	0.699
Mean blood glucose (mg/dL)	124.80(112.02, 144.80)	124.14(111.89, 145.88)	0.790
Coefficient of variation of blood glucose (%)	19.3(14.1, 27.8)	19.6(14.2, 28.2)	0.158
Hypertension, *n* (%)			0.504
0	3,192 (52.4)	1,388 (53.2)	
1	2,902 (47.6)	1,223 (46.8)	
Diabetes, *n* (%)			0.479
0	4,375 (71.8)	1855 (71.0)	
1	1719 (28.2)	756 (29.0)	
Heart failure, n (%)			0.591
0	4,633 (76.0)	1999 (76.6)	
1	1,461 (24.0)	612 (23.4)	
Myocardial infarction, *n* (%)			0.507
0	5,419 (88.9)	2,309 (88.4)	
1	675 (11.1)	302 (11.6)	
Malnutrition, *n* (%)			0.081
0	5,528 (90.7%)	2,337 (89.5%)	
1	566 (9.3%)	274 (10.5%)	
Feeding within 48 h of ICU admission, *n* (%)			0.502
0	265 (4.3)	122 (4.7)	
1	5,829 (95.7)	2,489 (95.3)	
Hospital stay before ICU admission (days)	7.19 (4.42, 12.92)	7.57 (4.43, 12.98)	0.441
ICU length of stay (days)	3.59(1.95, 7.46)	3.65(2.00, 7.62)	0.338
Duration of mechanical ventilation (h)	46.48(22.00, 95.97)	46.00(21.00, 95.39)	0.685
28-day ICU mortality, n (%)			1.000
0	5,425 (89.0)	2,325 (89.0)	
1	669 (11.0)	286 (11.0)	

The results indicated that the training and test sets were highly balanced in terms of demographic characteristics, laboratory parameters, and disease severity scores, with no statistically significant differences observed for any of the included variables between the two groups (all *p* > 0.05). Specifically, the median SOFA and APACHE II scores were identical between the two groups, at 5 and 17 points, respectively. Key clinical indicators, including partial pressure of oxygen, mean blood glucose, and lactate, were also evenly distributed between groups. In addition, the 28-day ICU mortality rate was 11.0% in both groups (*p* = 1.000), further confirming that the outcome risk distribution was well balanced after random splitting.

Supplementary Table S2 presents the VIF and tolerance values for all candidate variables. The mNUTRIC score showed evidence of notable multicollinearity (VIF = 8.4218; tolerance = 0.1187), which was considered clinically plausible because age, APACHE II score, and SOFA score are components of the mNUTRIC score and had also been included as individual candidate predictors. Therefore, the composite mNUTRIC score was excluded from subsequent feature selection to avoid redundant representation of the same clinical information. After exclusion of the mNUTRIC score, no evidence of significant multicollinearity was observed among the remaining candidate variables (all VIFs < 5 and tolerance values > 0.1).

### 3.2 Feature selection

Feature selection was performed using LASSO logistic regression combined with 10-fold cross-validation. The optimal regularization parameter (λ.1se = 0.01350446) was selected using the one-standard-error criterion (Figure 2A). Figure 2B illustrates the coefficient paths for different values of log(λ), with redundant coefficients of features shrinking to zero as λ increased. Ultimately, 9 core variables with non-zero coefficients were retained (Figure 2C), ranked by the absolute values of their coefficients, the selected variables were partial pressure of oxygen (PO₂), mean blood glucose, age, BMI, APACHE II score, hemoglobin, lactate, duration of mechanical ventilation, and urea nitrogen.

**Figure 2:**
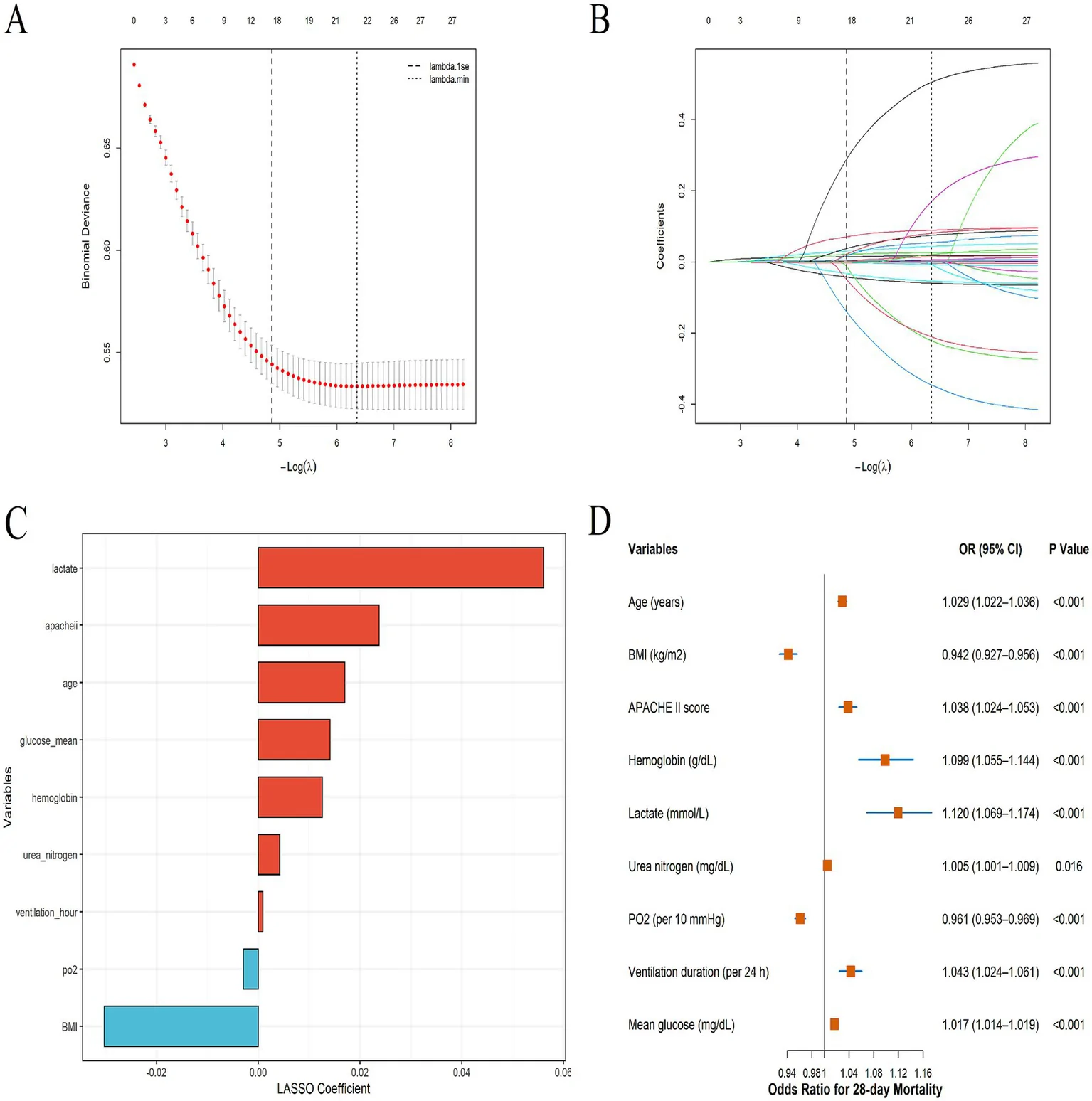
LASSO regression feature selection and multivariable logistic regression results. **(A)** LASSO regression cross-validation curve showing the variation in binomial deviance with log (λ); **(B)** LASSO regression coefficient path plot illustrating the trajectories of feature coefficients across different values of log (λ); **(C)** bar plot of non-zero LASSO coefficients, with the *x*-axis representing coefficient values and y-axis representing the 9 core variables retained at λ = λ1se; **(D)** forest plot of the multivariate logistic regression analysis.

These nine variables were subsequently included in a multivariable logistic regression analysis. To improve clinical interpretability, PO₂ and mechanical ventilation duration were modeled and reported per 10-mmHg and per 24-h increments, respectively. As shown in Figure 2D, all nine variables were significantly associated with the risk of 28-day ICU mortality (all *p* < 0.05). Higher age, APACHE II score, hemoglobin, lactate, urea nitrogen, mean blood glucose, and longer mechanical ventilation duration were associated with increased odds of 28-day mortality, whereas higher BMI and PO₂ were associated with decreased odds of mortality. These findings indicate that the nine features selected by LASSO were independently associated with 28-day mortality among critically ill patients with RFS. The resulting model was parsimonious and demonstrated favorable clinical interpretability.

### 3.3 Model development and evaluation

Three machine learning models (LR, XGBoost and RF) were developed using the 9 core variables and compared with traditional clinical scoring systems (Supplementary Table S3). Table 2 further presents the detailed performance metrics of each model. In the training set, the AUC values of the LR, RF, and XGB models were 0.823, 0.817, and 0.886, respectively. Among them, the XGB model showed the highest discriminative performance in the training set, suggesting a stronger fitting capacity for the training data. In the internal test set, the AUC values of the LR, RF, and XGB models were 0.821, 0.783, and 0.831, respectively. The overall discriminative abilities of the three models were comparable, indicating that all models achieved favorable predictive performance in internal validation. Although XGB yielded a slightly higher AUC in the internal test set, the improvement over LR was limited. Meanwhile, the LR model demonstrated high sensitivity (0.85) and negative predictive value (0.974) in the test set, showing robust overall performance and superiority over traditional scoring systems. These findings suggest that the LR model may have greater clinical utility for predicting 28-day ICU mortality risk.

**Table 2:** Performance comparison of machine learning models.

Algorithm	AUC	Sensitivity	Specificity	Youden index	Accuracy	PPV	NPV	F1 value
Training set								
LR	0.823	0.822	0.695	0.517	0.709	0.25	0.969	0.383
RF	0.817	0.807	0.725	0.532	0.734	0.266	0.968	0.4
XGB	0.886	0.812	0.79	0.601	0.792	0.322	0.971	0.462
Test set								
LR	0.821	0.85	0.683	0.533	0.701	0.248	0.974	0.384
RF	0.783	0.724	0.698	0.422	0.701	0.228	0.954	0.347
XGB	0.831	0.717	0.77	0.487	0.764	0.277	0.957	0.4

LR, logistic regression; RF, random forest; XGB, extreme gradient boosting; AUC, area under the curve; PPV, positive predictive value; NPV, negative predictive value.

### 3.4 External validation

To validate the generalizability of the optimized models, the MIMIC-III dataset and the hospital dataset were used as two external validation cohorts to assess the external predictive performance of the three models. As shown in Figure 3A, in the MIMIC-III external validation cohort, the AUC values of the LR, RF, and XGBoost models were 0.809, 0.583, and 0.762, respectively, with the LR model demonstrating the best discriminative performance. As shown in Figure 3B, in the hospital external validation cohort, the AUC values of the LR, RF, and XGBoost models were 0.731 (95% CI: 0.635–0.827), 0.727 (95% CI: 0.629–0.826), and 0.748 (95% CI: 0.651–0.844), respectively. Although the hospital validation cohort was relatively small, the overlapping confidence intervals suggested comparable discriminative performance among the three models.

**Figure 3:**
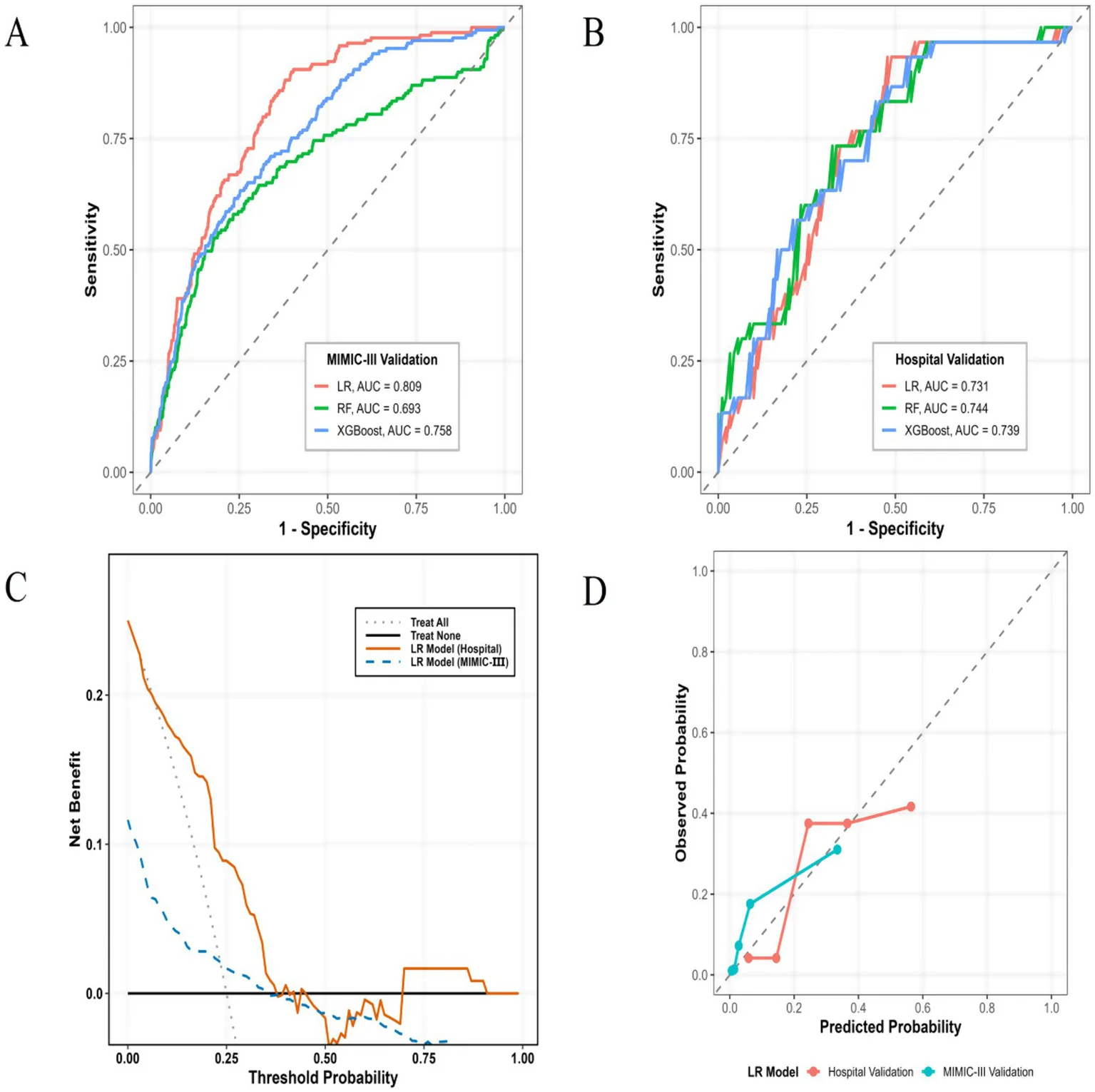
Performance assessment diagram of external validation models. **(A)** ROC curves in the MIMIC-III external validation cohort; **(B)** ROC curves in the hospital external validation cohort; **(C)** decision curve analysis of the LR model in the two external validation cohorts; **(D)** calibration curves of the LR model in the two external validation cohorts. LR, logistic regression; RF, random forest; XGB, extreme gradient boosting; AUC, area under the curve; CI, confidence interval.

Considering the performance across both external validation cohorts, the LR model showed relatively stable performance across datasets from different sources; therefore, its clinical utility and calibration performance were further evaluated. As shown in Figure 3C, decision curve analysis indicated that the LR model achieved a certain net benefit across part of the threshold probability range in both external validation cohorts, suggesting its potential clinical value for stratifying 28-day mortality risk among ICU patients. As shown in Figure 3D, the calibration curves showed an overall degree of agreement between the risks predicted by the LR model and the observed risks, although some calibration deviation remained. This deviation was more pronounced in the hospital external validation cohort, which may be related to differences in population characteristics, clinical workflows, and sample size across the external cohorts.

Taken together, the LR model maintained acceptable discriminative performance, a certain degree of clinical net benefit, and reasonable calibration performance in both external validation cohorts, indicating its favorable potential for external application.

### 3.5 Risk stratification and survival analysis

Based on the optimal cutoff value determined in the training set, patients were classified into high-risk and low-risk groups according to the predicted probabilities generated by the LR model, followed by Kaplan–Meier survival analysis. The results showed that, in the training cohort, patients in the high-risk group had a significantly lower probability of ICU survival than those in the low-risk group (*p* < 0.001, Figure 4A). Similar findings were also validated in the MIMIC-III external validation cohort, where patients in the high-risk group exhibited significantly poorer 28-day ICU survival outcomes (*p* < 0.001, Figure 4B). In the hospital external validation cohort, the survival curves also differed significantly between the high-risk and low-risk groups (*p* = 0.0011, Figure 4C).

**Figure 4:**
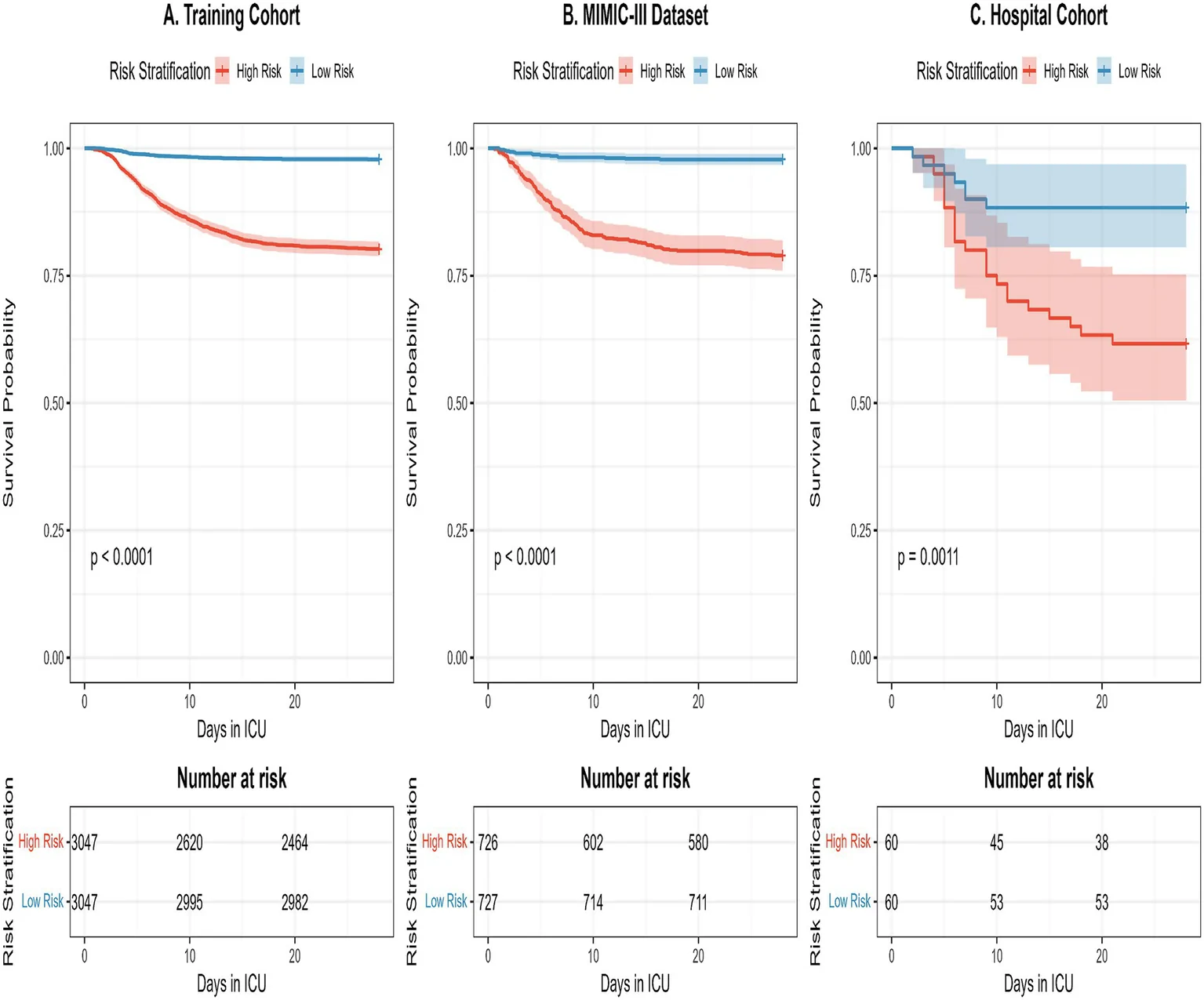
LR model-based risk-stratified survival analysis. **(A)** The 28-day ICU survival curves for the high- and low-risk groups in the training cohort; **(B)** the 28-day ICU survival curves for the high- and low-risk groups in the MIMIC-III external validation cohort; **(C)** the 28-day ICU survival curves for the high- and low-risk groups in the hospital external validation cohort. Red indicates the high-risk group, blue indicates the low-risk group, and the shaded areas represent the 95% confidence intervals. The numbers at risk at each time point are shown below the curves.

These results indicate that the LR model not only has favorable predictive ability for mortality risk but can also effectively distinguish RFS patients with different prognostic risk levels, thereby providing a basis for clinical risk stratification and early intervention.

### 3.6 Feature importance

To examine the contribution and direction of effect of each predictor on model output, SHAP analysis was performed based on the best-performing LR model. Figure 5A presents the SHAP summary plot, which visually illustrates the relationship between the values of each variable and the predicted risk. Figure 5B shows the ranking of variable importance. The results indicated that partial pressure of oxygen, mean blood glucose, and age were the top three key variables influencing the model’s prediction of 28-day ICU mortality risk, suggesting that oxygenation status, glycemic status, and physiological condition contributed substantially to risk discrimination by the model.

**Figure 5:**
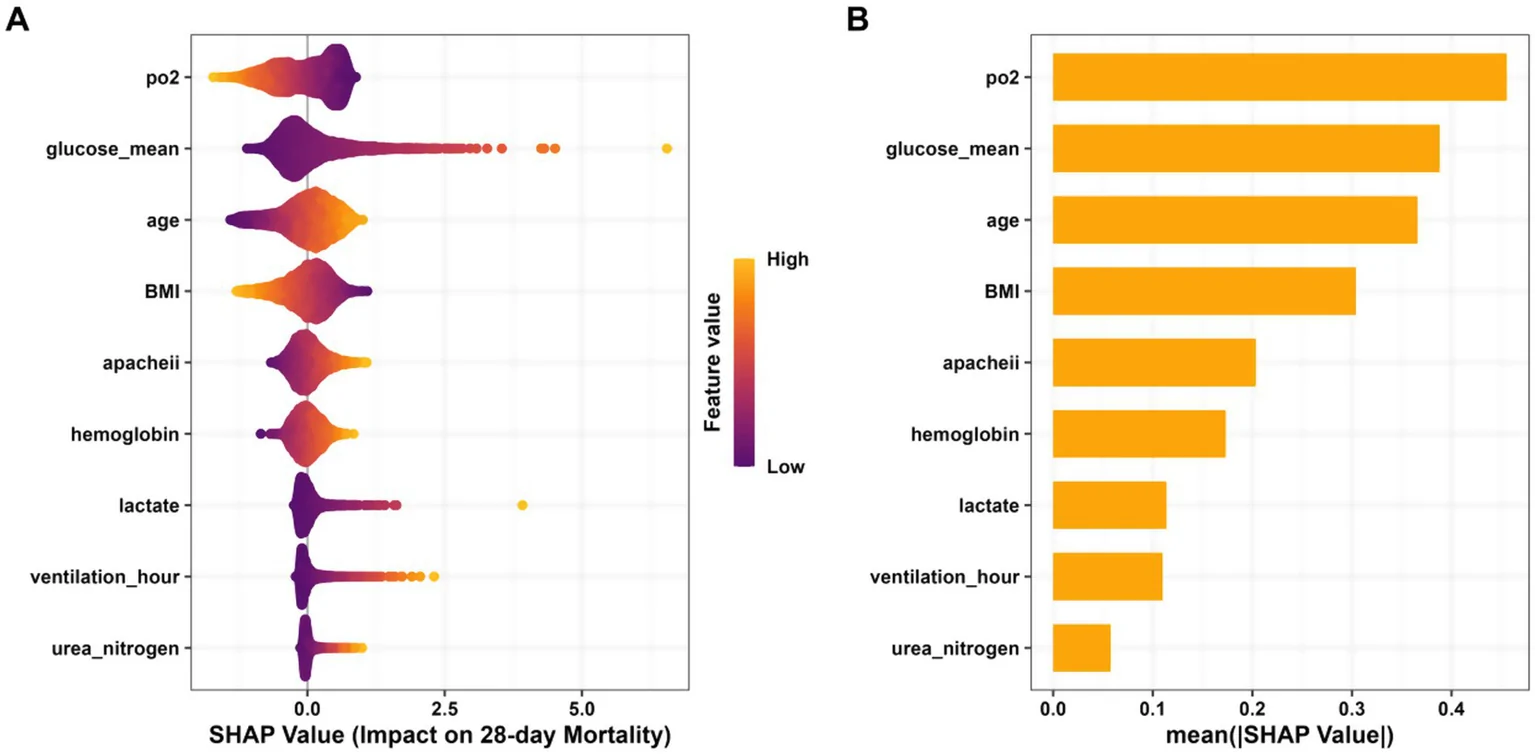
SHAP analysis results. **(A)** SHAP summary plot. Each point represents an individual patient sample, and the color indicates the magnitude of the variable value, with yellow representing higher values and purple representing lower values. SHAP values greater than 0 indicate that the variable contributes to a higher predicted mortality risk, whereas SHAP values less than 0 indicate that the variable reduces the predicted risk. **(B)** Feature importance ranking of the LR model.

Figure 6 reveals nonlinear relationships between most variables and the model-predicted risk. Specifically, age, APACHE II score, mean blood glucose, lactate, urea nitrogen, and duration of mechanical ventilation showed an overall increasing trend in SHAP values as their levels increased, whereas partial pressure of oxygen and BMI generally showed negative trends. These findings further support the directional effects of these variables in the model.

**Figure 6:**
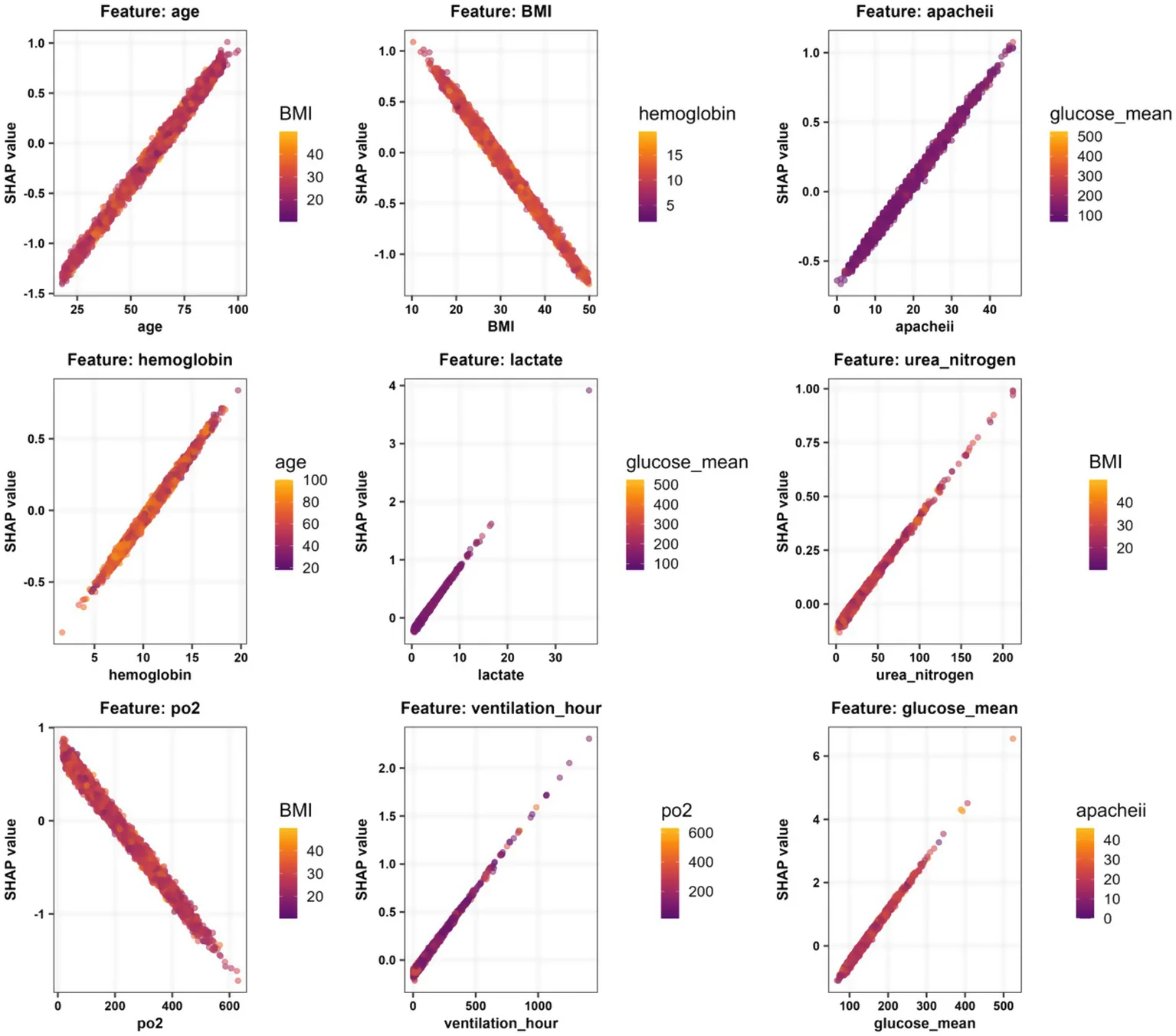
SHAP dependence plots: Each dependence plot shows how an individual feature affects the output of the prediction model.

### 3.7 Visualization of individualized risk prediction

To facilitate clinical application, we visualized the optimal logistic regression (LR) model. Figure 7 shows the nomogram of the LR model, which maps the core predictive variables onto point scores and estimates the probability of 28-day ICU mortality based on the total score, thereby providing an intuitive tool for rapid clinical risk assessment.

**Figure 7:**
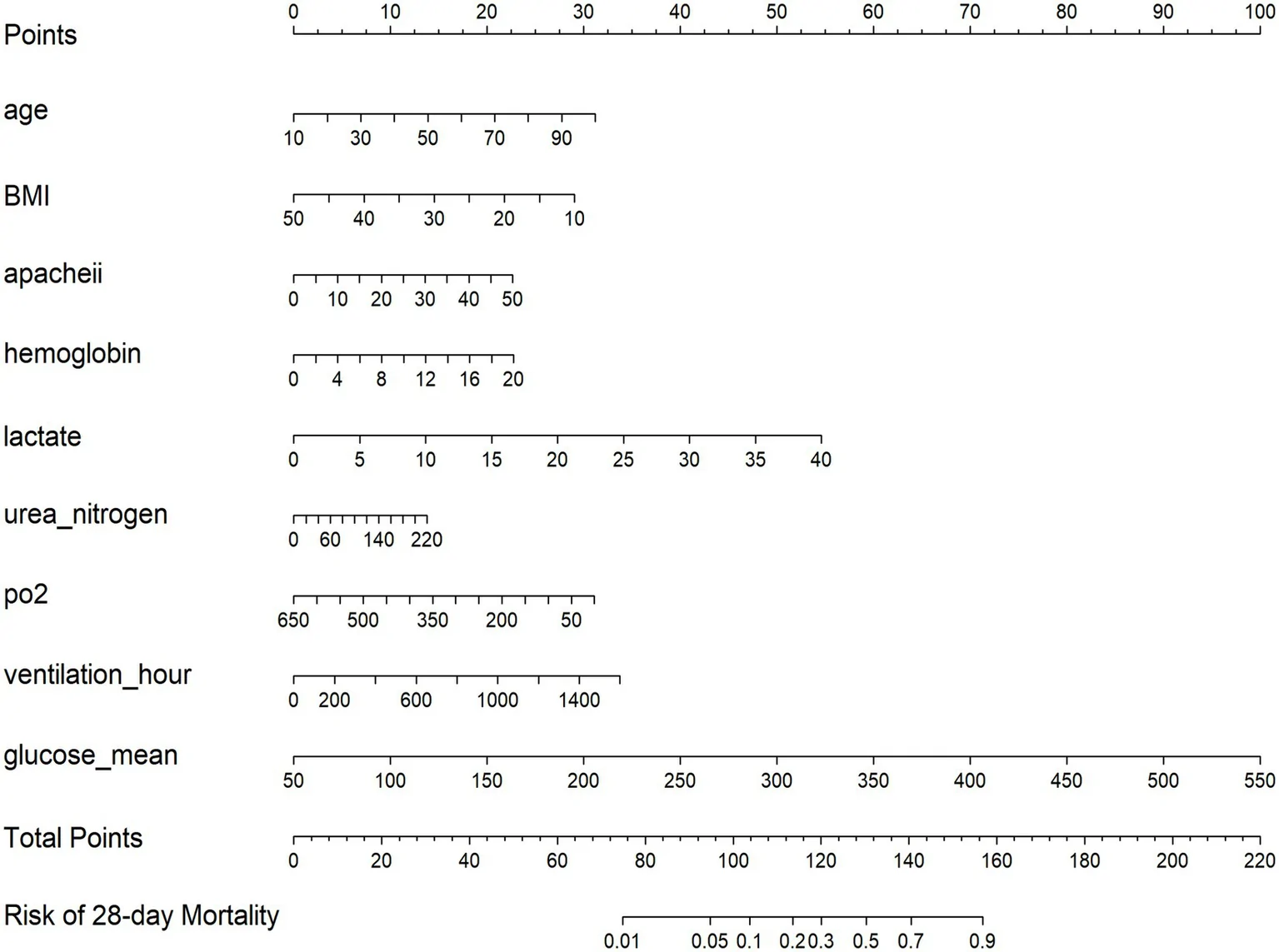
Nomogram of the LR model: The nomogram shows the point score assigned to each variable and the corresponding probability of 28-day ICU mortality based on the total score.

Based on this model, we developed an interactive R Shiny application.^1^ Users can input patient-specific indicators, including age, BMI, APACHE II score, hemoglobin, lactate, urea nitrogen, PO₂, duration of mechanical ventilation, and mean blood glucose, to obtain an individualized prediction of mortality risk. The application also displays the contribution of each variable to the predicted risk using a bar chart, with red indicating increased risk and blue indicating reduced risk. This tool provides an intuitive visualization of variable weights and supports the identification of high-risk patients and clinical decision-making regarding interventions (Figure 8).

**Figure 8:**
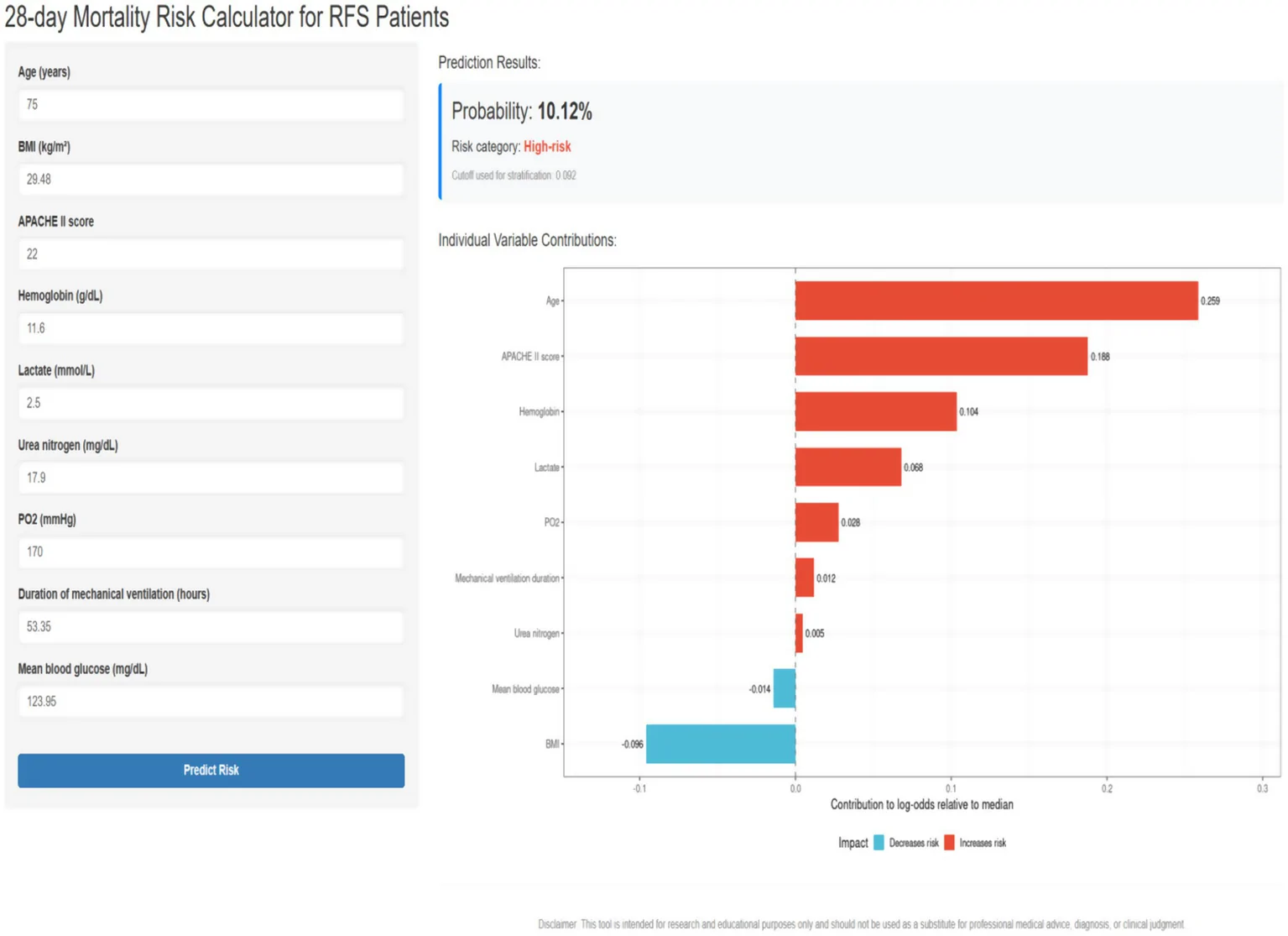
Example interface of the R Shiny application: The interface shows the predicted mortality risk after entering patient information, as well as the contribution of each variable to the predicted risk.

## 4 Discussion

Few previous machine learning studies have focused on predicting 28-day mortality among critically ill patients with refeeding syndrome. In this study, we developed and validated three predictive models using the MIMIC-IV, MIMIC-III databases and the local hospital, with logistic regression demonstrating superior predictive performance and clinical utility. To address the interpretability limitations commonly associated with machine learning models, SHAP analysis was applied to systematically evaluate the contribution of the 9 predictors, enhancing model transparency and supporting future clinical application.

The models showed stable and robust discriminative performance in both internal and external validation cohorts. In the test set, all three machine learning models significantly outperformed traditional clinical scoring system, indicating a superior ability to integrate multidimensional clinical data and capture complex relationships (23). Calibration and decision curve analyses further confirmed that the LR model provided accurate risk estimation and greater net clinical benefit across clinically relevant thresholds. External validation showed that the LR model achieved AUC values of 0.809 and 0.731, respectively, and maintained stable performance across heterogeneous cohorts from different sources, further supporting its favorable generalizability and potential clinical applicability.

The SHAP analysis identified the key predictors influencing the risk of 28-day mortality in critically ill patients with refeeding syndrome. Among them, partial pressure of oxygen, mean blood glucose, and age ranked as the top three contributors, suggesting that oxygenation status, glucose metabolism, and baseline physiological reserve may be critical determinants of short-term prognosis in this patient population. These indicators are routinely monitored parameters that can be rapidly obtained at the ICU bedside without additional testing costs, thereby facilitating the clinical implementation of the model.

In addition, variables such as BMI, APACHE II score, and hemoglobin also showed substantial predictive contributions. Notably, BMI was independently and inversely associated with 28-day mortality in the multivariable logistic regression analysis (OR = 0.942, 95% CI: 0.927–0.956). This finding is consistent with the “obesity paradox” reported in critically ill and mechanically ventilated populations, in which overweight or moderately obese patients may exhibit better short-term survival than patients with a lower or normal BMI (24, 25). Greater nutritional reserves and metabolic buffering capacity may help patients tolerate the abrupt metabolic and catabolic stress associated with nutritional reintroduction. However, this interpretation remains hypothetical, because BMI cannot distinguish adipose tissue from lean body mass, and the retrospective observational design of this study precludes causal inference. Together with APACHE II score and hemoglobin, these findings suggest that the model captures multiple prognostic dimensions, including nutritional reserve, disease severity, and oxygen-carrying capacity.

The core mechanism underlying refeeding syndrome involves the physiological response to rapid metabolic changes following nutritional restoration (26). The AuSPEN 2025 consensus statement further emphasizes that the identification and management of RFS should focus on the comprehensive assessment of metabolic disturbances, electrolyte abnormalities, and clinical complications during nutritional rehabilitation (27). Within this pathological context, the association between PO₂ and prognosis may primarily reflect an imbalance between oxygen supply and demand, as well as impaired respiratory function during refeeding. Hypophosphatemia is one of the typical manifestations of RFS and can affect cellular energy metabolism and muscle function. Previous reviews have indicated that severe hypophosphatemia may impair diaphragmatic contractility and lead to acute respiratory failure, tracheal intubation, or an increased need for mechanical ventilation (28). Meanwhile, RFS-related volume overload, impaired myocardial function, and electrolyte disturbances may further aggravate cardiopulmonary decompensation, making patients more susceptible to decreased oxygenation and an increased risk of death (29).

The predictive role of mean blood glucose may be related to carbohydrate loading and the insulin response after refeeding. When nutrition is reintroduced, increased glucose intake stimulates insulin secretion and promotes the intracellular shift of glucose, electrolytes, and water (30). In critically ill patients, insulin resistance under stress conditions, stress-induced hyperglycemia, and glycemic fluctuations may further exacerbate metabolic imbalance. Previous studies have shown that increased glycemic variability is associated with a higher risk of mortality in critically ill patients (31), and recent evidence also suggests that stress-induced hyperglycemia is closely related to adverse outcomes in ICU patients (32).

The effect of age may be attributable to reduced organ reserve, a higher prevalence of malnutrition, a greater burden of comorbidities, and diminished compensatory capacity in response to metabolic stress among older patients. A study focusing on older adults noted that RFS carries specific clinical risks in elderly patients, and its management requires comprehensive attention to nutritional status, vitamin deficiency, electrolyte disturbances, and abnormalities in fluid balance (33). Therefore, in critically ill patients with RFS, age is not merely a demographic variable but may also represent the patient’s overall physiological reserve and resilience to stress.

Therefore, the variables identified by the model in this study reflect key pathological processes underlying short-term adverse outcomes in critically ill patients with RFS from three dimensions: oxygenation status, glucose metabolic regulation, and baseline physiological reserve. These findings are highly consistent with the metabolic remodeling, oxygenation impairment, and decline in organ reserve involved in the development and progression of RFS.

Previous studies have developed risk prediction models for RFS in critically ill patients (34, 35), neurocritical patients (36), general hospitalized patients (37), postoperative colorectal cancer patients (38), and patients with severe acute pancreatitis (39). However, no studies have specifically focused on predicting 28-day ICU mortality in critically ill patients with RFS, and dedicated tools for short-term prognostic assessment in this population remain lacking. In addition, many prior studies were limited by small sample sizes, which constrained predictive performance and generalizability.

This study was based on large-scale public healthcare databases, using MIMIC-IV data from 2008 to 2019 as the internal training and validation cohort, and MIMIC-III data from 2001 to 2007 and local hospital data as two external validation cohorts. The stability and generalizability of the model were evaluated across three dimensions: different time periods, different databases, and different geographic settings. Methodologically, this study integrated LASSO-based feature selection, machine learning model development, SHAP-based interpretability analysis, nomogram construction, and the development of an interactive R Shiny tool. These procedures followed established standards for clinical prediction model development and may help improve the robustness, interpretability, and clinical translation potential of the model.

## 5 Limitations

This study has several limitations. First, the retrospective observational design of this study, which used public databases and a local hospital cohort, may have introduced selection and information biases, and some prognostically relevant factors may not have been captured. In particular, detailed and standardized information regarding the initial caloric target or infusion rate, daily feeding advancement, feeding interruptions, and the agents, routes, and doses used for electrolyte replacement was not consistently available. In addition, the frequency of electrolyte monitoring varied according to individual clinical conditions rather than following a uniform study protocol. These differences in nutritional support and electrolyte management may have influenced the development and clinical course of RFS and could not be fully adjusted for in the present analysis.

Second, although this study performed external validation using the MIMIC-III dataset and a local hospital dataset, the number of external validation cohorts remains limited. In particular, the hospital external validation cohort had a relatively small sample size and a limited number of outcome events, which may have increased the uncertainty of model performance estimates and resulted in wider confidence intervals. Although the model maintained acceptable discrimination in this cohort, further validation using larger, multicenter cohorts is required to obtain more precise estimates of model performance. Moreover, differences may exist across data sources in terms of population characteristics, clinical workflows, variable recording methods, nutritional management, and follow-up quality. Therefore, the generalizability of the model across broader regions and different healthcare systems requires further validation.

Third, the model developed in this study is a predictive model intended only to assist clinical risk assessment. It cannot establish causal relationships between the predictors and mortality outcomes, nor can it directly replace clinical judgment or be used to formulate specific treatment strategies.

Finally, prospective and multicenter clinical validation has not yet been conducted in this study. Future studies are needed to further evaluate the effectiveness, stability, and clinical utility of the model across different healthcare institutions and real-world clinical settings.

## 6 Conclusion

We developed and validated an interpretable LR model for predicting the risk of 28-day ICU mortality in critically ill patients with refeeding syndrome. The model demonstrated favorable predictive performance and risk stratification ability, and may assist clinicians in the early identification of high-risk patients while providing a reference for individualized management.

## Data Availability

The raw data supporting the conclusions of this article will be made available by the authors, without undue reservation.

## Footnotes

1
https://rfs28day-predictor.shinyapps.io/Final_App/
