# Supplementary material for: Construction and validation of a 28-day mortality prediction model for refeeding syndrome in critically ill patients
Source: Front Nutr. 2026 Sep 25;13:1888609. doi: 10.3389/fnut.2026.1888609 (PMC13650927; doi:10.3389/fnut.2026.1888609)
Supplement: Supplementary file 1 [file Table_1.DOCX]

| **Table S1** Univariate analysis results | | |
| --- | --- | --- |
| Variables | OR_95CI | P-value |
| Age (years) | 1.039 (1.032–1.045) | <0.001 |
| Gender, n (%) | 0.733 (0.623–0.863) | <0.001 |
| BMI (kg/m²) | 0.954 (0.941–0.967) | <0.001 |
| Ethnicity, n (%) | 1.397 (0.882–2.215) | <0.001 |
| Asian | 1.409 (1.040–1.908) | <0.001 |
| Black | 1.219 (0.772–1.925) | <0.001 |
| Latinos | 1.516 (1.265–1.815) | <0.001 |
| Other | 1.145 (1.118–1.174) | <0.001 |
| SOFA score | 0.967 (0.946–0.989) | 0.004 |
| GCS score | 1.075 (1.063–1.087) | <0.001 |
| APACHE II score | 1.397 (0.882–2.215) | <0.001 |
| mNUTRIC score | 1.399 (1.339–1.461) | <0.001 |
| Magnesium (mmol/L) | 0.546 (0.444–0.673) | <0.001 |
| Phosphorus (mmol/L) | 1.320 (1.229–1.417) | <0.001 |
| Potassium (mmol/L) | 0.935 (0.799–1.095) | 0.406 |
| Hemoglobin (g/dL) | 1.083 (1.045–1.121) | <0.001 |
| Total calcium (mmol/L) | 1.070 (0.976–1.173) | 0.152 |
| Lactate (mmol/L) | 1.198 (1.148–1.251) | <0.001 |
| Creatinine (mg/dL) | 1.215 (1.149–1.285) | <0.001 |
| Urea nitrogen (mg/dL) | 1.023 (1.020–1.027) | <0.001 |
| Partial pressure of carbon dioxide (mmHg) | 1.006 (0.999–1.012) | 0.111 |
| pH | 0.041 (0.019–0.092) | <0.001 |
| Partial pressure of oxygen (mmHg) | 0.994 (0.993–0.995) | <0.001 |
| Activated partial thromboplastin time (s) | 1.008 (1.005–1.011) | <0.001 |
| International normalized ratio | 1.218 (1.117–1.327) | <0.001 |
| Mean blood glucose (mg/dL) | 1.021 (1.019–1.024) | <0.001 |
| Coefficient of variation of blood glucose (%) | 1.016 (1.012–1.021) | <0.001 |
| Hypertension, n (%) | 0.858 (0.730–1.009) | 0.064 |
| Diabetes, n (%) | 1.002 (0.839–1.198) | 0.979 |
| Heart failure, n (%) | 1.731 (1.457–2.056) | <0.001 |
| Myocardial infarction, n (%) | 1.120 (0.875–1.435) | 0.373 |
| Malnutrition, n (%) | 0.498 (0.363–0.683) | <0.001 |
| Feeding within 48 h of ICU admission, n (%) | 0.858 (0.730–1.009) | 0.064 |
| Hospital stay before ICU admission (days) | 0.978 (0.969–0.987) | <0.001 |
| ICU length of stay (days) | 1.036 (1.026–1.046) | <0.001 |
| Duration of mechanical ventilation (h) | 1.003 (1.002–1.003) | <0.001 |
| *Note：*nivariate logistic regression analysis of factors associated with 28-day mortality in critically ill patients with refeeding syndrome. | | |
