# Supplementary material for: Construction and validation of a 28-day mortality prediction model for refeeding syndrome in critically ill patients
Source: Front Nutr. 2026 Sep 25;13:1888609. doi: 10.3389/fnut.2026.1888609 (PMC13650927; doi:10.3389/fnut.2026.1888609)
Supplement: Supplementary file 2 [file Table_2.DOCX]

| **Table S2** Multicollinearity Table of Variables | | |
| --- | --- | --- |
| Variables | VIF | Tolerance |
| Age | 2.5999 | 0.3846 |
| BMI | 1.1459 | 0.8726 |
| SOFA score | 1.1617 | 0.8608 |
| GCS score | 1.0361 | 0.9652 |
| APACHEⅡ score | 2.83 | 0.3534 |
| mNUTRIC score | 8.4236 | 0.1187 |
| Magnesium | 1.1845 | 0.8443 |
| Phosphorus | 1.4147 | 0.7068 |
| Potassium | 1.3076 | 0.7648 |
| Hemoglobin | 1.3867 | 0.7211 |
| Total calcium | 1.1428 | 0.875 |
| Lactate | 1.3057 | 0.7659 |
| Creatinine | 1.8103 | 0.5524 |
| Urea nitrogen | 2.0995 | 0.4763 |
| Partial pressure of carbon dioxide | 1.903 | 0.5255 |
| pH | 2.4307 | 0.4114 |
| Partial pressure of oxygen | 1.4088 | 0.7098 |
| Activated partial thromboplastin time | 1.1116 | 0.8996 |
| International normalized ratio | 1.1199 | 0.893 |
| Mean blood glucose | 1.6834 | 0.594 |
| Coefficient of variation of blood glucose | 1.314 | 0.7611 |
| Mean blood glucose | 1.6834 | 0.594 |
| Hypertension | 1.2468 | 0.8021 |
| Diabetes | 1.4517 | 0.6888 |
| Heart failure | 1.3244 | 0.7551 |
| Myocardial infarction | 1.0965 | 0.912 |
| malnutrition | 1.116 | 0.8961 |
| Feeding within 48 h of ICU admission | 1.0783 | 0.9274 |
| Hospital stay before ICU admission (days) | 1.3589 | 0.7359 |
| ICU length of stay | 3.2813 | 0.3048 |
| Duration of mechanical ventilation | 2.8589 | 0.3498 |
| *Note：*Multicollinearity among candidate variables was assessed using the variance inflation factor (VIF) and tolerance. The VIF and tolerance values for each variable are presented. | | |
