# Supplementary material for: Construction and validation of a 28-day mortality prediction model for refeeding syndrome in critically ill patients
Source: Front Nutr. 2026 Sep 25;13:1888609. doi: 10.3389/fnut.2026.1888609 (PMC13650927; doi:10.3389/fnut.2026.1888609)
Supplement: Supplementary file 3 [file Table_3.DOCX]

| **Table S3** Traditional Scoring Performance Table | | | | | | | | |
| --- | --- | --- | --- | --- | --- | --- | --- | --- |
| Algorithm | AUC | Sensitivity | Specificity | Youden Index | Accuracy | PPV | NPV | F1 value |
| Training set |  |  |  |  |  |  |  |  |
| only SOFA | 0.392 | 0.985 | 0.019 | 0.005 | 0.879 | 0.891 | 0.141 | 0.936 |
| Only APACHEII | 0.356 | 6e-04 | 1.000 | 6e-04 | 0.110 | 1.000 | 0.110 | 0.001 |
| SOFA+APACHEII | 0.646 | 0.666 | 0.576 | 0.242 | 0.656 | 0.927 | 0.175 | 0.775 |
| Test set |  |  |  |  |  |  |  |  |
| only SOFA | 0.418 | 0.986 | 0.025 | 0.010 | 0.881 | 0.892 | 0.175 | 0.936 |
| Only APACHEII | 0.358 | 4e-04 | 0.997 | -0.003 | 0.110 | 0.500 | 0.109 | 9e-04 |
| SOFA+APACHEII | 0.636 | 0.647 | 0.563 | 0.210 | 0.638 | 0.923 | 0.164 | 0.761 |
| External validation 1 |  |  |  |  |  |  |  |  |
| only SOFA | 0.380 | 0.988 | 0.012 | -0.001 | 0.874 | 0.884 | 0.111 | 0.933 |
| Only APACHEII | 0.365 | 0.013 | 0.929 | -0.059 | 0.119 | 0.571 | 0.110 | 0.024 |
| SOFA+APACHEII | 0.660 | 0.018 | 0.994 | 0.012 | 0.132 | 0.958 | 0.118 | 0.035 |
| External validation 2 |  |  |  |  |  |  |  |  |
| only SOFA | 0.325 | 1.000 | 0.000 | 0.000 | 0.750 | 0.750 | NA | 0.857 |
| Only APACHEII | 0.247 | 0.000 | 1.000 | 0.000 | 0.250 | NA | 0.250 | 0.000 |
| SOFA+APACHEII | 0.762 | 0.378 | 0.900 | 0.278 | 0.508 | 0.919 | 0.325 | 0.535 |
| AUC: area under the curve; PPV, positive predictive value; NPV, negative predictive value.  *Note：*The predictive performance of SOFA, APACHE II, and the combination of SOFA and APACHE II was evaluated using the area under the receiver operating characteristic curve (AUC), sensitivity, specificity, Youden index, accuracy, positive predictive value (PPV), negative predictive value (NPV), and F1 score in the training set, test set, and two external validation cohorts. | | | | | | | | |
